# When is accuracy off-target?

**DOI:** 10.1038/s41398-021-01479-4

**Published:** 2021-06-18

**Authors:** Melissa D. McCradden

**Affiliations:** 1 Bioethics Department, The Hospital for Sick Children, Toronto, ON Canada; 2 Division of Clinical & Public Health, Dalla Lana School of Public Health, Toronto, ON Canada

**Keywords:** Psychiatric disorders, Scientific community

**Dear Editor,**

Medicine is a complex system of continuously evolving knowledge. Patients characterized within this knowledge system are heterogenous, and have contextual complexity evading even the most robust algorithm. Due to this complexity and evolution, even the very best artificial intelligence (AI; more precisely, machine learning, ML) systems when making present and future predictions will inevitably be wrong some of the time. Empowering clinicians to recognize and reject incorrect predictions is of utmost importance.

‘Explainability’ (a general term referring to methods that enable a user to ‘understand’ an ML prediction) has received a lot of attention both inside and outside of the ML field. Some ethical AI guidelines have proposed explainability as a core principle akin to autonomy or beneficence [1] and stressed its purported importance to accountable decision-making [2]. But a recent paper in *Translational Psychiatry* reveals that explainability may not live up to its ethical ideal.

Jacobs et al. [3] conducted an elegant study exploring an important and under-examined issue: the independent influence of explanations on decision-making. By simulating an ML model designed to recommend antidepressants (top 5 expert-determined drugs per patient scenario) the authors explored the impact of the accuracy of recommendations and their explanations on 220 clinicians’ drug choices across five patient descriptions. Scenarios were systematically varied to establish differences across correct/incorrect choices as a function of the type of explanation presented, including: recommendations alone, placebo (‘based on ICD-10 codes’), feature-based explanations (highlighting patient-specific features) and heuristic-based explanations (reflecting general drug-related knowledge). Clinicians’ judgment compared with their baseline (no recommendation) was negatively affected by incorrect recommendations accompanied by explanations, with the strongest effect occurring for features-based explanations.

These compelling findings are important additions to the accumulating evidence highlighting the risks of over-trust (defined herein as reliance on incorrect ML predictions) in the context of clinical decisions informed by ML [4–6]. The implications that clinicians’ own accuracy may be negatively impacted by incorrect, explainable ML predictions contradicts the idea that explainability can effectively mitigate ‘black box’ concerns [1, 2]. As a matter of patient safety, further research is essential to understanding how explainability might introduce novel risks to carefully consider whether or when it should be used at all.

Yet, such comparisons may not always be as simple as quantifying individual decisions as ‘correct’ or ‘incorrect.’ As Jacobs et al. [3] note, dropout risk is but one factor in choosing in antidepressant and the same authors have stressed the importance of shared decision-making [7]. As we move this field forward it is worth considering: when and how is accuracy important for clinical decision-making?

Initiating antidepressant treatment is a shared decision between patients and clinicians, better guided recently by the accumulated evidence from a plethora of randomized controlled trials [8, 9]. Antidepressants have differing side effect profiles and quasi-distinct neurochemical mechanisms of actions. Because of these differences, clinicians are advised to recommend specific drugs based on the patient’s predominant or most troubling symptoms (e.g., psychosis), severity of symptoms, atypical features, possibility of overdosing, and concurrent medical problems [8]. Patients may also have particular preferences and wishes regarding the tolerability of specific side effects. Yet, these preferences are notably complicated by direct-to-consumer marketing, which can influence patients to request medications that are less well studied, more expensive, and potentially inappropriate [10]. Moreover, some ideal decisions are not achievable due to inequities in access to healthcare and health insurance—would we consider a clinician to have chosen incorrectly for prescribing a more affordable medication?

To the contrary, consider accuracy in the context of diagnosis. A diagnosis is generally made according to the presence of signs, symptoms, and other biomarkers that indicate the presence of a medical condition. Patient preferences are not central to determining a diagnosis as they are for making treatment decisions. In this case, clinician accuracy is scored on their ability to detect the true presence of a disease just as it would be for an ML model.

It is particularly interesting that Jacobs et al. [3] found that feature-based explanations with incorrect predictions were more compelling than heuristic-based explanations and placebo. Clinicians, as noted, make judgements integrating medical evidence with patient-level factors to identify options in the patient’s best interests. The extent to which ML models appear to do the same may increase the perception that the model is not just operationalizing a single value (dropout risk), but instead is replicating the whole clinical decision-making process. By being very clear on which values are operationalized by the ML model and considering them as distinct from values underpinning clinical judgment, we can move toward complementary—rather than competitive—conceptualizations of ML-inclusive decision-making.

A hint that this may be happening is in Jacobs and et al. particularly intriguing finding that clinicians with the most knowledge about ML relied on it less but were more confident and ranked it more useful than their less-experienced peers [3]. Perhaps in recognizing the operationalization of one value (dropout risk) as but one axis informing decision-making, they can appreciate the information supplied by the model while not allowing it to subsume the larger clinical goal (to help the patient).

Guided by moral commitments to patient autonomy and best interests, clinical decision-making incorporates medical evidence as but one factor in a larger picture in which accuracy is but one metric. ML contributes to the evidence base by operationalizing a particular axis of decision-making. The key to preventing over-reliance is perhaps not in providing explanations, but in compartmentalizing these axes with clinicians accountable for the moral goal not of accuracy, but of helping patients.
